# A Dual Challenge: *Coxiella burnetii* Endocarditis in a Patient with Familial Thoracic Aortic Aneurysm—Case Report and Literature Review

**DOI:** 10.3390/jcm13237155

**Published:** 2024-11-26

**Authors:** Alina-Ramona Cozlac, Caius Glad Streian, Marciana Ionela Boca, Simina Crisan, Mihai-Andrei Lazar, Mirela-Daniela Virtosu, Adina Ionac, Raluca Elisabeta Staicu, Daniela-Carmen Dugaci, Adela Emandi-Chirita, Ana Lascu, Dan Gaita, Constantin-Tudor Luca

**Affiliations:** 1 Department VI Cardiology-Cardiovascular Surgery, “Victor Babes” University of Medicine and Pharmacy of Timișoara, Eftimie Murgu Square No. 2, 300041 Timisoara, Romania; alina-ramona.cozlac@umft.ro (A.-R.C.); simina.crisan@umft.ro (S.C.); lazar.mihai@umft.ro (M.-A.L.); adina.ionac@umft.ro (A.I.); dan.gaita@umft.ro (D.G.); constantin.luca@umft.ro (C.-T.L.); 2 Institute for Cardiovascular Diseases of Timisoara, “Victor Babes” University of Medicine and Pharmacy of Timisoara, G. Adam Str. No. 13A, 300310 Timisoara, Romania; boca.marciana@cardiologie.ro (M.I.B.); daniela.cozma@umft.ro (M.-D.V.); raluca.staicu@umft.ro (R.E.S.); dugaci.daniela@cardiologie.ro (D.-C.D.); lascu.ana@umft.ro (A.L.); 3 Advanced Research Center of the Institute for Cardiovascular Diseases, “Victor Babes” University of Medicine and Pharmacy of Timișoara, Eftimie Murgu Square No. 2, 300041 Timisoara, Romania; 4 Department VI Cardiology Internal Medicine and Ambulatory Care, Prevention and Cardiovascular Recovery, “Victor Babeș” University of Medicine and Pharmacy of Timisoara, Eftimie Murgu Square No. 2, 300041 Timisoara, Romania; 5 Doctoral School Medicine-Pharmacy, “Victor Babes” University of Medicine and Pharmacy of Timișoara, Eftimie Murgu Square No. 2, 300041 Timisoara, Romania; 6 Centre of Genomic Medicine, Genetics Discipline, “Victor Babeș” University of Medicine and Pharmacy of Timișoara, 300041 Timisoara, Romania; adela.chirita@umft.ro; 7 Department III Functional Sciences—Pathophysiology, “Victor Babes” University of Medicine and Pharmacy of Timișoara, Eftimie Murgu Square No. 2, 300041 Timisoara, Romania; 8 Centre for Translational Research and Systems Medicine, “Victor Babes” University of Medicine and Pharmacy of Timișoara, Eftimie Murgu Square No. 2, 300041 Timisoara, Romania

**Keywords:** thoracic aortic aneurysms, *ACTA2* gene variant, *Coxiella burnetii*, infective endocarditis, chronic Q fever

## Abstract

**Background/Objectives**: Thoracic aortic aneurysms (TAAs) are potentially life-threatening medical conditions, and their etiology involves both genetic and multiple risk factors. *Coxiella burnetii* endocarditis is one of the most frequent causes of blood culture-negative infective endocarditis (BCNIE) in patients with previous cardiac surgery. Our review aims to emphasize the importance of genetic testing in patients with thoracic aortic aneurysms but also the importance of additional testing in patients with suspected endocarditis whose blood cultures remain negative. The reported case has a history of acute DeBakey type I aortic dissection that developed during her second pregnancy, for which the Bentall procedure was performed at that time. Ten years after the surgery, the patient started developing prolonged febrile syndrome with repeatedly negative blood cultures, the serological tests revealing the presence of an infection with *Coxiella burnetii*. Considering her family history and the onset of her aortic pathology at a young age, genetic tests were performed, disclosing a missense variant in the actin alpha-2 (*ACTA2*) gene in heterozygous status. **Methods**: For a better understanding of both conditions, our research was conducted in two directions: one reviewing the literature on patients with *Coxiella burnetii* BCNIE and the other focusing on patients who had a familial thoracic aortic aneurysm (FTAA) due to the *ACTA2* variant. This review incorporates studies found on PubMed and ResearchGate up to August 2024. **Conclusions**: BCNIE represents a condition with several diagnostic challenges and may lead to severe complications if timely treatment is not initiated. Also, diagnosing an FTAA requires genetic testing, enabling better follow-up and management.

## 1. Introduction

Thoracic aortic aneurysms (TAAs) involve the dilation of the vessel caliber, with the aortic diameter thresholds adapting to physical measurements, such as height and weight [1,2]. In addition to the etiology involving inflammatory and infectious diseases or risk factors, such as hypertension and hypercholesterolemia, genetic factors implicated in the physiopathology of vascular wall syndromes have gained significant importance in recent years, leading to improved classification and management of aortic aneurysms [3,4]. The latest AHA Guidelines for the Diagnosis and Management of Aortic Disease recommend routine genetic testing of patients diagnosed with thoracic aortic aneurysm at a young age or having features associated with specific syndromes; additionally, DNA sequencing should be performed on known at-risk relatives of individuals with positive results [2]. FTAAs are caused by their association with variants of either extracellular matrix protein function or vascular smooth muscle proteins [1]. TAAs can be divided into two main subtypes depending on multiorgan damage, the branches being referred to as syndromic or non-syndromic. The syndromic ones include diseases affecting both the vascular system and other connective tissue abnormalities, the most notable examples of this group being Loeys-Dietz syndrome, Marfan syndrome, or vascular Ehlers-Danlos syndrome. Conversely, non-syndromic TAAs are defined by their impact solely on the cardiovascular system [5].

The pathogenic genes involved in the pathway of FTAAs affect proteins that ensure the integrity of the vascular wall, particularly vascular smooth muscle cells. Multiple genes can determine a familial pattern of thoracic aortic aneurysms, most of them having autosomal dominant inheritance, with the highest studied and prominently featured in the academic field being *ACTA2*, *MYH11*, *MYLK*, *PRKG1*, and those involved in the TGF-β pathway [6,7]. Pathogenic variants of the *ACTA2* gene, which encodes α-actin, are some of the most frequent etiologies of FTAAs. Alpha-actin expresses almost half of the total proteins in smooth muscle cells (up to 40%), thus making it the most representative protein at this level [6]. In addition to cardiovascular disorders, pathogenic variants of the *ACTA-2* gene predispose patients to other forms of vascular pathologies, including Moyamoya-like occlusions that can lead to early onset of cerebrovascular events or occlusions of internal carotid arteries or intracranial aneurysms [8,9,10].

On the other hand, the reported case is one with complex particularities, with several medical specialties being involved and both the patient’s genetic and infectious pathology leading to consequences in the cardiovascular system. The infectious disease that the patient contracted during the last few years was an infection with *Coxiella burnetii*. This micro-organism is the causative agent of Q fever, being intracellular gram-negative bacteria, and is usually transmitted directly from animals, like goats or sheep, or indirectly by ticks [11,12]. It has been repeatedly indicated as a potential biological threat because it can be transmitted through inhalation, it has a low infective dose, and it has environmental stability [13,14]. The acute onset of the disease manifests as a high fever and flu-like symptoms, for example, a nonproductive cough, but because of the non-specific symptoms, the treatment is not always administered on time, allowing the Q fever to turn into a chronic disease [15,16]. The only method of preventing the disease is through immunization achieved by the Q-vax vaccine, but it is currently only administered in Australia [17].

The most common manifestation of chronic Q fever is blood culture-negative infective endocarditis, but in some cases, the onset form may be represented by hepatitis, osteomyelitis, febrile illness lasting up to fifty days, and even neurological pathology, characterized by encephalitis, meningitis, or peripheral neuropathy [18,19].

The specific treatment of *Coxiella burnetii* infection is the administration of Doxycycline for 14 days for the acute infection, but in the case of chronic Q fever, a combination of Doxycycline and Hydroxychloroquine should be administered for up to 18 months [20,21]. According to the 2023 ESC Guidelines for the management of endocarditis, blood culture-negative infective endocarditis caused by *Coxiella burnetii* should be treated with Doxycycline 200 mg/24 h and Hydroxychloroquine 200–600 mg/24 h for a minimum of 18 months [22].

## 2. Materials and Methods

The main information sources of the studies examined in this review are PubMed and ResearchGate. The studies were selected based on the inclusion criteria described above, with the selected articles that resemble the reported case including only those published since January 2000. Firstly, the studies selected for the first part of the review, namely patients presenting with BCNIE, had to meet the following criteria: case reports published between January 2000 and August 2024 that include patients currently being diagnosed positive for *Coxiella burnetii* endocarditis but having an aortic valve replacement in their medical history. Article types other than case reports published before 2000 and studies involving non-human participants were excluded. For the second part of the review, the studies included case reports published between January 2000 and August 2024 that describe patients with the *ACTA2* gene causing a familial thoracic aortic aneurysm; the exclusion criteria remained the same as those applied in the first section of the review.

The following keywords were used to maximize search accuracy: “*Coxiella burnetii* infection”, “Q fever”, “mechanical aortic valve”, and “blood culture-negative infective endocarditis” for the first part of the review, and “familial thoracic aortic aneurysm”, “*ACTA2* variant/mutation”, “aortic dissection”, and “*ACTA2* gene”, for the second part. The keywords were selected to improve research accuracy and capture the intricate pathologies described in our case report. The MeSH function on PubMed was used to increase the specificity of the research by using combinations of the keywords. The discovered articles were added to the Zotero application, with the ones identified as duplicates being removed. We used Microsoft 365 (Office) software, Microsoft Corporation, Redmond, WA, USA. 

Next-generation sequencing (NGS) was performed in the Center for Genomic Medicine, Timișoara, for the patient and one daughter in a panel of 174 genes, using the Illumina TruSight Cardio Sequencing Panel kit and a MiSeq Illumina sequencing platform (Illumina, San Diego, CA, USA). End-to-end bioinformatics algorithms were implemented, using Burrows-Wheeler Aligner (BWAAligner), SAMtools, Genome Analysis Toolkit (GATK-Variant Caller-Broad Institute of MIT and Harvard, Cambridge, MA, USA) and Annovar (as described elsewhere) [23]. Alignment of the sequenced fragments was performed on the human reference genome GRCh37. A tertiary data analysis was performed at the level of current knowledge using online databases and aggregators, including Varsome, ClinVar, gnomAD, DECIPHER, UCSC Genome Browser, OMlM, DGV, and Ensembl. Variants were classified according to the 2015 ACMG guideline [24].

## 3. Results

The reported case describes a 45-year-old woman known to have multiple cardiovascular conditions and a positive family history, namely her mother died at 36 years old of unknown causes (a possible spontaneous carotid artery dissection). The patient developed acute aortic dissection DeBakey type I during her second pregnancy, which was treated surgically with a Bentall procedure in 2006 by replacing the aortic valve and the ascending aorta with a valved composite graft with re-implantation of the coronary arteries into the graft.

The clinical evolution of the patient was favorable for the first 15 years, followed only by chronic anticoagulant treatment of Acenocumarol 4 mg, adjusted according to the INR test. In December 2021, she started developing prolonged febrile syndrome, which was extensively investigated and treated with antibiotics several times. For two years, the patient was hospitalized in various clinics, and sets of blood cultures were performed repeatedly, all with negative results. After all the investigations were carried out, the established diagnoses were blood culture-negative infective endocarditis on the mechanical aortic valve, anti-neutrophil cytoplasmic antibody (ANCA)-associated systemic vasculitis, chronic glomerulonephritis (nephritic syndrome form) with splenic infarction, and diet-controlled type 2 diabetes.

**Figure 1:**
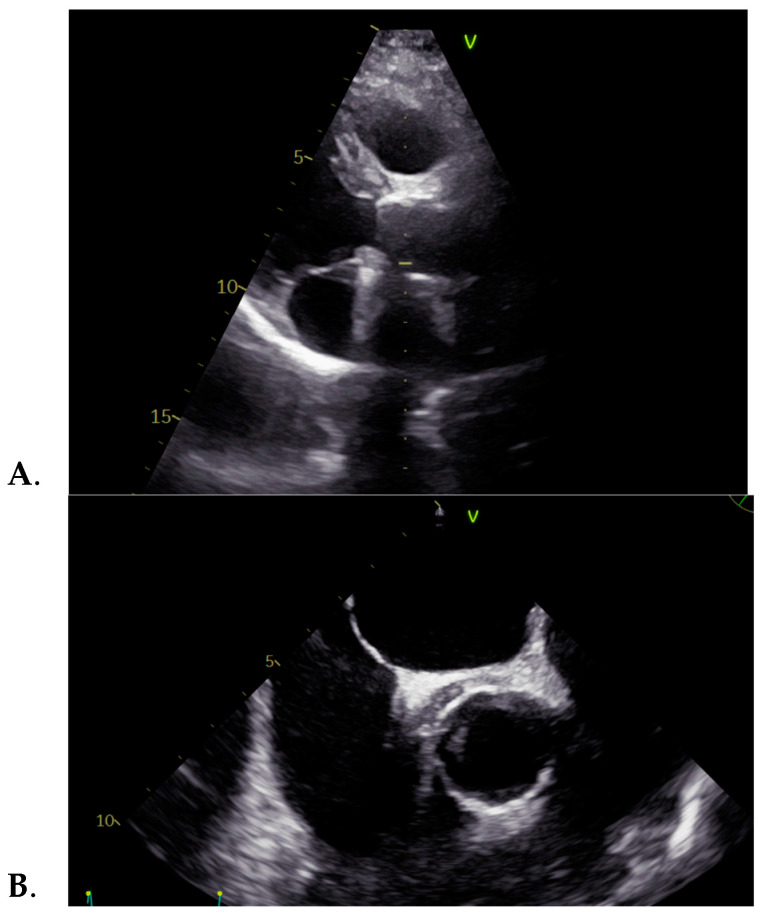
(**A**) Transthoracic and (**B**) transesophageal echocardiographies showing an echo-dense mass surrounding the ascending aorta.

In June 2024, the patient was admitted to our clinic for suspected intermittent febrile syndrome with subfebrile onset for about 3 months, accompanied by vertigo. The medical examination revealed normal vital signs and pale skin, and the cardiac examination highlighted a systolic murmur, heard loudest in the aortic area with a closing click. The ECG was normal. The pathological blood samples collected at admission revealed an inflammatory syndrome, i.e., highly elevated ESR and C-reactive protein, with positive procalcitonin. The transthoracic echocardiography showed an apparently normal mechanical prosthesis in the aortic position, mild intraprosthetic aortic regurgitation, mild mitral regurgitation, mild functional tricuspid regurgitation, mild secondary pulmonary hypertension, and a roughly 12 mm echo-dense mass surrounding the ascending aorta, also visible on the transesophageal echocardiography, as seen in Figure 1. Transesophageal echocardiography provides additional information on heart morphology, usually being used for a better view of the left atrium and the left atrial appendage in order to detect thrombi, but in our case, it was used for a better view of the aortic valve [25,26].

On the second day after admission, the patient experienced a subfebrile episode, which prompted the collection of blood cultures, but the results were negative. Consequently, more specific investigations were performed, and serological tests for infective endocarditis of diverse etiology were acquired, including IgG and IgM antibodies for *Mycoplasma pneumoniae*, *Bartonella henselae*, *Chlamydophila pneumoniae*, *Brucella* spp., *Legionella pneumophila*, and *Coxiella burnetii*. All results were negative, except for *Coxiella burnetii* IgG and IgM phase I and phase II antibodies, and the results of both the phase I and phase II immunofluorescent assay (IFA) highlighted increased antibody titers, as seen in Table 1. Likewise, the PCR for *Coxiella burnetii* in the blood by molecular hybridization with an amplification test also revealed a positive result. Therefore, the diagnosis of acute onset of chronic Q fever as a disease was confirmed, the targeted therapy with Hydroxychloroquine 200 mg tid and Doxycycline 100 mg bid was initiated, and treatment will continue to be administered for 18 months. A relevant reference is the patient’s travel history; starting from 2021, the patient had not traveled abroad, and no direct link could be made to a potential source of infection, such as domestic animals. However, from 2006 to 2011, the patient traveled to several African countries for about 3 months/year, and starting in 2012, she traveled several times to Switzerland and Germany.

**Figure 2:**
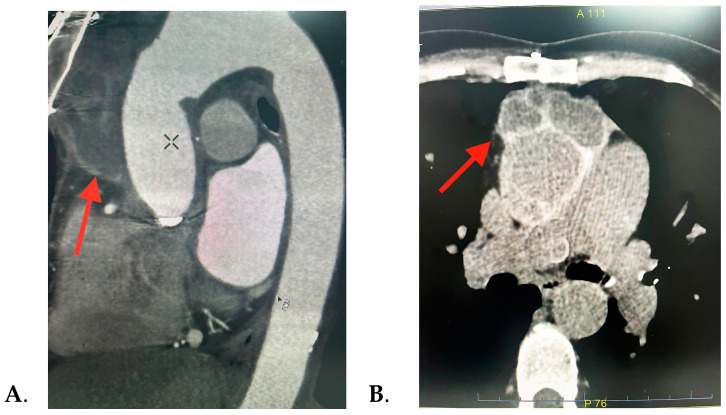
Thorax CT scan images showing the periaortic fluid accumulation (red arrow): (**A**) sagittal and (**B**) axial sections.

Consequently, with the echocardiographic images revealing a significant peri-aortic mass, it was decided to perform further investigations. The thorax CT scan detected a multiloculated right semi-circumferential fluid accumulation with iodophilic walls that extends from the level of the aortic valve along the aortic prosthesis to the anterior mediastinum, suggesting a peri-aortic abscess, as seen in Figure 2. Pericardial and pleural fluid collection and mediastinal adenopathies were also found.

**Figure 3:**
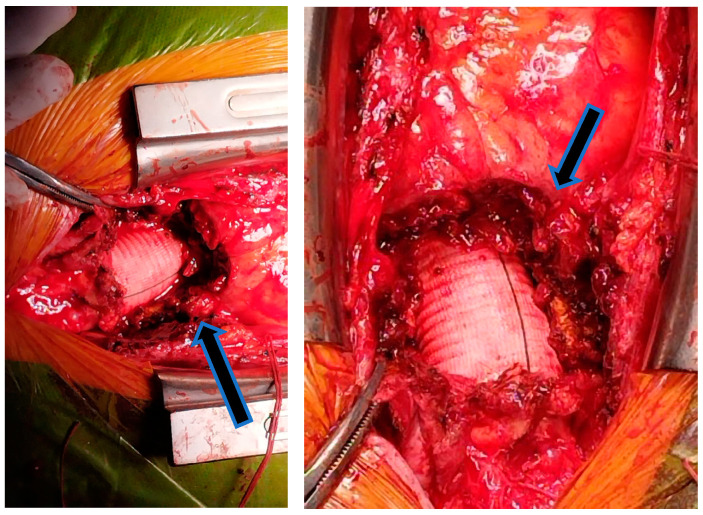
Intraoperative images showing the periaortic fluid accumulation (black arrow).

The preliminary diagnosis was *Coxiella burnetii* infective endocarditis complicated with mediastinal abscess; thus, a multidisciplinary team, consisting of a cardiologist, cardiovascular surgeon, infectionist, and intensive care physician, decided that the optimal management in this case would be a surgical redo operation with antibiotic protection. The surgery involved the removal of the periaortic fluid mass, with the macroscopic examination suggesting a liquefied chronic hematoma that can be seen in Figure 3. The bacteriological examination of the collected biological products, pericardial fragment, periprosthetic and subprosthetic tissue, and clot fragment revealed no bacterial or fungal growth on the inoculated culture media.

The post-operative evolution was favorable, and the investigations continued with genetic tests. Genetic testing for a familial aortopathy was proposed considering the positive family history of early cardiovascular-related death, the patient’s mother having passed away at the age of 36, possibly due to a spontaneous carotid artery dissection. The patient’s onset of the aortic pathology manifested as thoracic aortic dissection at the age of 27, which also constituted one of the factors that prompted the suspicion of a hereditary condition, thereby leading to genetic testing. A missense variant (variant NM_001613.4:c.773G>A, NP_001604.1:p.(Arg258His)) was detected in heterozygous status in the *ACTA2* gene located on chromosome 10q23.31. This variant was identified in several individuals with FTAAs, segregated by phenotype. The variant was classified with pathogenic significance according to the American College of Medical Genetics and Genomics (ACMG) guideline [24]. As a result, the suspicion of a genetic disease was confirmed, establishing the diagnosis of familial thoracic aortic aneurysm 6, part of a non-syndromic FTAA cluster. In some cases, the pathogenic variant of *ACTA2* is associated with intracranial aneurysms and Moyamoya-like cerebrovascular disease, so the patient will be undergoing a cranial CT scan [27]. Subsequently, her two daughters are proposed to be genetically tested and will have follow-up echocardiographic examinations.

To provide a better perspective on the patient’s clinical status, paraclinical data, personal and family medical histories, and the treatment have been summarized in Table 2.

The patient was discharged with a good general condition and the following diagnosis: blood culture-negative infective endocarditis with *Coxiella burnetii*, liquefied chronic periprosthetic hematoma (surgically treated), status post-Bentall operation, normofunctional double disc mechanical prosthesis in the aortic position, mild mitral regurgitation, mild functional tricuspid regurgitation, mild secondary pulmonary hypertension, NYHA I chronic heart failure with preserved ejection fraction, diet-controlled type 2 diabetes, ANCA-associated systemic vasculitis, chronic glomerulonephritis (nephritic syndrome form), enlarged spleen with subcapsular infarction, and mild normochromic normocytic anemia, and the following treatment being recommended at home: Hydroxychloroquine 200 mg/tid, Doxycycline 100 mg bid, Acenocoumarol 4 mg (dose controlled by INR test in order to maintain a value between 2.5–3.5), diuretics (association between Furosemide/Spironolactone 20/50 mg/day), and a probiotic. The specific antibiotic treatment for *Coxiella burnetii* BCNIE with Hydroxychloroquine and Doxycycline will be administered for 18 months, according to the 2023 ESC Guidelines for the management of endocarditis. This approach is tailored to the patient’s clinical course, the prolonged progression of febrile syndrome with multiple recurrences, and the potential complications that could be associated with mediastinal spread of the infection [22]. The only side effects reported by the patient regarding the specific medication for *Coxiella burnetii* were insomnia and tinnitus.

## 4. Discussion

In order to perform an accurate review starting with the reported case, which included two major significant pathologies, both a genetically inherited disease, the *ACTA2* variant gene leading to a familial thoracic aortic aneurysm and dissection, as well as a rare infectious disease, namely blood culture-negative infective endocarditis on a mechanical aortic valve, the research on the medical literature started by looking for the coexistence of both pathologies, but it failed to identify any patient similar to the case presented above.

Therefore, our research was oriented toward searching separately for the two intricate pathologies, the review encompassing medical articles describing, on one hand, blood culture-negative infective endocarditis with *Coxiella burnetii* on a mechanical aortic valve and, on the other hand, studies presenting patients having familial thoracic aortic aneurysm and/or dissection due to the *ACTA2* disease-causing variant.

Firstly, in the interest of achieving similarities between the described patient’s infectious disease with cardiovascular implications and the patients included in the chosen studies, we identified articles published in the medical literature between 2000 and August 2024 available on PubMed and ResearchGate. The inclusion criteria that the selected articles had to meet were patients with a medical history including a surgery replacing the native aortic valve with a prosthetic one; patients diagnosed with *Coxiella burnetii* blood culture-negative infective endocarditis; studies including clear diagnosis and management parameters and having an explicit outcome for the patients; articles published in the target period of time; and studies defined as case reports. The exclusion criteria were studies published before 2000; studies having qualities other than case reports; articles that did not include accurate patient management details; and studies including non-human participants. The research strategy was designed using keywords and phrases closely relevant to the reported case; thus, the keywords included “*Coxiella burnetii* infection”, “Q fever”, “mechanical aortic valve”, and “blood culture-negative infective endocarditis”.

There were four identified case reports describing *Coxiella burnetii*-associated blood culture-negative infective endocarditis on a prosthetic aortic valve, as seen in Table 3.

The most frequently described symptom in these patients was fever, and, in most of the cases, febrile symptomatology started about a month before the presentation. In our patient, fever was present for about two and a half years, with periods of remission, subfebrility, or aggravation episodes.

The treatment in two of the cases was exclusively medical, one patient benefited from surgical intervention, and one case was managed through both medical and surgical therapy. One case was treated with a special antibiotic, as Afrasiabian et al. [30] described intolerance to Doxycycline, and thus the antibiotic treatment chosen instead was levofloxacin. Krol et al. [31] also preferred the administration of monotherapy with Doxycycline for 5 months, leading to a favorable result and an improved patient outcome. The treatment preferred by Bozza et al. [28] was the administration of Doxycycline 100 mg/bid in association with Hydroxychloroquine 200 mg/tid for 24 months, with a long-term follow-up of the patient, which proved the efficacy of the chosen antibiotics through a decreasing trend in the serological tests. This combination of antibiotics was also chosen for the patient treated in our clinic, being well tolerated by the patient, without fever remissions.

The characteristics of the *Coxiella burnetii* infection selected to compare with the patient’s evolution described in our case report with the literature data represent only a subset of the features associated with this complex disease. *Coxiella burnetii* is defined as an intracellular bacterium causing a zoonotic disease transmitted from animals like sheep, cattle, or goats to humans, with ticks being the main reservoir in nature. The transmission is usually performed through aerosols, but vertical, transplacental, or even nosocomial infection is also possible. In humans, the infection may present many forms, including an asymptomatic course or progression through two distinct phases, acute and chronic disease. Acute infection can manifest as febrile symptomatology or flu-like onset, but it may also appear as hepatitis, pneumonia, or other manifestations. The presence and duration of fever were included in the comparative characteristics evaluated in the patients analyzed in this review. The most common manifestation of chronic Q fever is endocarditis; however, hepatitis, ophthalmological or neurological disorders, and infections of vascular grafts may also be present as forms of the disease.

In addition, a second part of the research was the comparison of the patient’s evolution regarding the aortic disease caused by the *ACTA2* variant with the patients described in the chosen articles. The research among the medical literature provided five case reports published between 2000 and August 2024 that describe patients having a thoracic aortic aneurysm with a positive family history and the *ACTA2* variant, as seen in Table 4. The inclusion criteria were patients presenting any type of variant of the *ACTA2* gene expressing vascular smooth muscle cells; studies having the quality of case reports; and studies providing clear evidence of patient management. On the other hand, the exclusion criteria were studies involving non-human participants; studies published before the year 2000; and studies with characteristics other than case reports. The examination was conducted among the studies found on PubMed and ResearchGate.

The prevalence of the targeted aortic pathology was found to be higher among men, with 57.1% of the patients described in the selected studies being males. Similarly, the *ACTA2* disease-causing variant genes were discovered to be more frequent among males with aortic disease. Concerning the average age of patients with familial thoracic aortic aneurysm, the oldest patient experiencing acute onset of the condition was 41 years old, as described by Hoffjan et al. [32], while the youngest was just 15 years old, namely the study published by Ware et al. [33] presenting the twins who developed acute aortic dissection at the same time. In the patients’ family history, cases carrying the mutant gene were identified after genetic counseling and testing because they did not experience any clinical manifestation of the disease. Similar to the reported data in the literature, our patient’s clinical onset was very early; the acute aortic dissection occurred at the age of 27.

Among the included patients, only two of them presented a notable phenotypic manifestation, namely congenital mydriasis in the twins described by Ware et al. [33]. This clinical expression was also described in the specialized literature as being strongly associated with a particular missense variant in arginine 179 in the *ACTA2* gene [5].

The complexity of the reported case arises from the early onset of the aortic disease, the patient being 7 months pregnant when she presented at the hospital for acute aortic dissection. This circumstance is consistent with the documented data regarding the evolution of pregnancies in females with the *ACTA2* variant [34]. Thus, Hoffjan et al. [32] described two patients with a history of physiological pregnancy before being diagnosed with mild aortic dilation and acute aortic dissection. The academic literature describes a powerful connection between an underlying aortic disease and an aortic dissection associated with pregnancy. Fluctuations in pregnancy hormone levels, along with the hemodynamic stress experienced by pregnant women, can exacerbate a subjacent aortic condition, particularly during the third trimester [35].

Furthermore, genetic counseling should be the standard practice in all the cases outlined above. This management step is crucial for families where one member has already been identified for the *ACTA2* disease-causing variant, as this gene is associated with an increased risk of vascular diseases, as seen in every presented study. Considering the autosomal dominant transmission of the *ACTA2* gene, each child of an affected parent has a 50% chance of inheriting the variant; thus, the two daughters of the patient presented in our case report will undergo genetic counseling to facilitate future cardiovascular follow-up and ensure that any potential complication can be managed in time. If a child is found to be a hereditary carrier, management strategies should focus on regular cardiovascular monitoring through echocardiograms or CT scans and implementing lifestyle changes that minimize stress on the cardiovascular system. This might include controlling blood pressure and avoiding heavy lifting or other risk factors, such as smoking. Pregnancy will be monitored closely as it adds additional stress to the aorta. In some cases, preventive surgical options might be discussed if imaging indicates a high risk of vascular events. In addition to medical information, genetic counseling addresses the emotional impact of a genetic diagnosis, providing resources and support for the psychological aspects of living with an inherited condition. Discussing the options for preimplantation genetic or prenatal testing of the *ACTA2* variant is included in the genetic counseling.

**Table 4:** Patients presenting the *ACTA2* gene and their characteristics.

No.	First Author/Year/Reference	No. of Patients/Gender/Age	Medical History of Aortic Disease	Family History of Aortic Disease	Clinical Features	Healthy Pregnancy
1.	Hoffjan et al., 2011 [32]	3 patients a. F, 38 b. F, 37 c. M, 41	a. Mild aortic dilation b. Acute aortic dissection c. Thoracic aortic aneurysm	a. 1 brother had ascending aorta aneurysm and died at 46 years old; 1 brother and the father had thoracic aortic aneurysm b. 1 brother died at 29 years old from acute aortic dissection c. no data available	Not described	a. 2 healthy pregnancies b. 2 healthy pregnancies c. -
2.	Keravnou et al., 2018 [36]	1/M/30	Type A aortic dissection	Father had aortic root and ascending aorta aneurysm (Bentall procedure performed) Mother had ascending aortic dilation	Not described	-
3.	Marutani et al., 2023 [37]	1/M/15	Extensive dissection from the ascending aorta to the common iliac artery	Genetic tests not performed	Not described	-
4.	Ware et al., 2014 [33]	1/M/17	Recurrent aortic dissection, severe aortic regurgitation	Twin brother had aortic dissection	Congenital mydriasis (both twins)	-
5.	Delsart et al., 2021 [38]	1/F/29	DeBakey type I aortic dissection	2 siblings had acute aortic dissection Mother died at 49 years old from type B aortic dissection	Not described	No pregnancies

## 5. Conclusions

To summarize, a familial thoracic aortic aneurysm is a condition with a high mortality rate if it is not properly diagnosed and monitored over time. These findings should encourage more frequent genetic testing in patients with aortic aneurysms, especially those with significant familial medical history or an early onset of the aortic disease. Additionally, a condition of particular importance but meaningful diagnostic and therapeutical challenges is blood culture-negative infective endocarditis. Rare causes should be investigated, and serological testing for infectious agents such as *Coxiella burnetii* ought to be considered. The management of a patient with both a genetic variant already manifested through a cardiovascular complication and a concurrent infectious disease is very particular due to the complexity of the case and requires a comprehensive, multidisciplinary approach focused on surveillance and proactive care. Therefore, regular cardiovascular imaging, blood pressure monitoring, and infection status assessment are essential to detect early signs of complications. Genetic counseling and family screening may also be beneficial for identifying at-risk relatives, enabling timely follow-up.

## Figures and Tables

**Table 1:** Serology of *Coxiella burnetii* antibodies.

Serology	Value
IgG phase I antibodies	>1:4096
IgM phase I antibodies	1:2024
IgG phase II antibodies	>1:4096
IgM phase II antibodies	1:1024

**Table 2:** Significant characteristics of the patient.

Clinical Manifestation	Family History of Aortic Disease	History of Cardiovascular Surgery	Blood Samples	Echocardiography Findings	Fever History	Treatment
-prolonged febrile syndrome -vertigo -pale skin -closing click in the aortic area	Mother died at 36 y (spontaneous carotid artery dissection)	Bentall procedure (for acute DeBakey type I aortic dissection)	-elevated ESR -elevated C-reactive protein -positive procalcitonin -negative blood cultures -elevated phase I and phase II IgG and IgM	-mechanical prosthesis in aortic position -12 mm echo-dense mass surrounding the ascending aorta	2 years	Medical: -antibiotics: Doxycycline (100 mg/bid) and Hydroxychloroquine (200 mg/tid) -Acenocumeral 4 mg -Furosemide/Spironolactone 20/50 mg/day Surgical: removal of the periaortic hematoma

**Table 3:** Patients presenting *Coxiella burnetii* blood culture-negative endocarditis and their characteristics.

No	First Author/Year/Reference	No. of Patients/Gender/Age	History of Cardiovascular Surgery	Fever History	Surgical or Medical Treatment
1	Bozza et al., 2023 [28]	1/M/55	Aortic valve replacement (aortic regurgitation and aneurysm)	1 month	Medical: Doxycycline (100 mg/bid) and Hydroxychloroquine (200 mg/tid)
2	Deyell et al., 2006 [29]	1/M/31	Open valvulotomy for congenital aortic stenosis + mechanical aortic replacement for severe aortic regurgitation	Not described	Surgical: Aortic root replacement of the ascending aorta and aortic valve replacement
3	Afrasiabian et al., 2024 [30]	1/F/67	Aortic valve replacement	1 month	Medical: Levofloxacin-intolerance of Doxycycline
4	Krol et al., 2008 [31]	1/F/43	Aortic valve replacement (bicuspid aortic valve)	Fever history, but no time described	Both; medical: Doxycycline monotherapy

## Data Availability

All data are mentioned in the manuscript.
